# The rexinoid, bexarotene, prevents the development of premalignant lesions in MMTV-erbB2 mice

**DOI:** 10.1038/sj.bjc.6604320

**Published:** 2008-03-25

**Authors:** Y Li, Y Zhang, J Hill, H-T Kim, Q Shen, R P Bissonnette, W W Lamph, P H Brown

**Affiliations:** 1 Breast Center, Departments of Medicine and Molecular and Cellular Biology, Baylor college of Medicine, One Baylor Plaza, Houston, TX 77030, USA; 2 Department of Retinoid Research, Ligand Pharmaceuticals Inc., San Diego, CA 92121, USA

**Keywords:** rexinoid, bexarotene, prevention, breast cancer, ER negative, MMTV-erbB2

## Abstract

Retinoids, vitamin A analogues that bind to retinoic acid receptor (RAR) or retinoid X receptor (RXR), play important roles in regulating cell proliferation, apoptosis, and differentiation. Recently, RXR-selective ligands, also referred to as rexinoids, have been investigated as potential chemopreventive agents for breast cancer. Our previous studies demonstrated that the rexinoid bexarotene significantly prevented ER-negative mammary tumourigenesis with less toxicity than naturally occurring retinoids in animal models. To determine whether bexarotene prevents cancer at the early stages during the multistage process of mammary carcinogenesis, we treated MMTV-erbB2 mice with bexarotene for 2 or 4 months. The development of preinvasive mammary lesions such as hyperplasias and carcinoma-in-situ was significantly inhibited. This inhibition was associated with reduced proliferation, but no induction of apoptosis. We also examined the regulation of a number of rexinoid-modulated genes including critical growth and cell cycle regulating genes using breast cell lines and mammary gland samples from mice treated with rexinoids. We showed that two of these genes (DHRS3 and DEC2) were modulated by bexarotene both *in vitro* and *in vivo*. Identification of these rexinoid-modulated genes will help us understand the mechanism by which rexinoid prevents cancer. Such rexinoid-regulated genes also represent potential biomarkers to assess the response of rexinoid treatment in clinical trials.

Despite earlier diagnosis and treatment advances, breast cancer remains the leading health threat to women (Jemal *et al*, 2005). Moreover, the annual incidence rate of breast cancer in the United States has increased steadily over the last two decades, only falling in the last few years (Edwards *et al*, 2002). This high incidence rate has prompted strong interest in breast cancer prevention. The past chemoprevention studies have focused heavily on antioestrogen drugs, which include selective oestrogen receptor modulators (tamoxifen and raloxifene) and aromatase inhibitors. These agents are effective in reducing the risk of ER-positive breast cancer. However, they all act against oestrogen signalling pathways and are unable to prevent ER-negative breast cancer, which accounts for one-third of all breast cancers and has a particularly poor prognosis (Cuzick *et al*, 2003). Recently, a number of novel chemopreventive agents targeting oestrogen-independent signalling pathways have been developed. Among them, retinoids have been shown to prevent the development of ER-negative mammary tumours in animal models (Wu *et al*, 2000, 2002a, 2002b). In addition, clinical data show that synthetic retinoid fenretinide significantly reduced the occurrences of both contralateral and ipsilateral breast cancer incidence in premenopausal women (Veronesi *et al*, 1999, 2006). More importantly, fenretinide was observed to reduce secondary tumours irrespective of the hormone receptor status of the primary cancer (Menard *et al*, 2001), suggesting that retinoids have potential chemopreventive effect towards ER-negative breast cancer.

Retinoids are vitamin A analogues that bind nuclear retinoid receptors, retinoic acid receptor (RAR) and/or retinoid X receptor (RXR). The ligand-bound receptors form dimeric complexes that interact with DNA at specific retinoid-responsive elements and regulate the transcription of target genes that control cellular proliferation, differentiation, and apoptosis (Yang *et al*, 1999). Based on this nuclear receptor specificity, retinoids can be categorised as RAR-selective, RXR-selective, or as pan-agonists, which bind both RAR and RXR. Previous studies demonstrated that 9cRA, a naturally occurring retinoid that binds both RAR and RXR, is effective in preventing ER-negative mammary tumorigenesis in C3(1)-SV40 T-antigen transgenic mice; however this pan-agonist had significant toxicity (Wu *et al*, 2000). TTNPB, an RAR-selective retinoid, was highly toxic and was not able to prevent breast cancer in mouse models (Wu *et al*, 2002a). In contrast, the RXR-selective retinoid bexarotene (LGD1069, Targretin) was effective in preventing ER-negative mammary tumorigenesis in mice, and had less toxicity than 9cRA and the RAR-selective retinoids (Wu *et al*, 2002a). Thus, RXR-selective retinoids (also referred to rexinoids) represent more effective and more tolerable agents for the prevention of breast cancer. Subsequently, we reported that bexarotene significantly delayed ER-negative mammary tumour formation in MMTV-erbB2 and SV40 T-antigen transgenic mice. Both mouse models develop ER-negative mammary tumours, suggesting that the well-tolerated rexinoid is an effective agent for the prevention of ER-negative breast cancer.

Although significant progress has been made towards understanding the RAR/RXR mediated signalling pathway, the mechanism by which bexarotene suppresses tumourigenesis is still poorly understood. Mammary carcinogenesis in humans is a chronic and multistage process that proceeds through normal epithelium, atypical hyperplasia, ductal carcinoma *in situ* (DCIS), and invasive breast cancer (Beckmann *et al*, 1997). Similarly, tumours developed in MMTV-erbB2 transgenic mice also arise through a multistage process (Kosanke *et al*, 2004). Bexarotene inhibits cell proliferation in normal human mammary epithelial cells (Wu *et al*, 2006), suggesting that it will prevent tumour formation at the early stages during the chronic multistage carcinogenesis. In this study, we investigated the ability of bexarotene to prevent the development of premalignant lesions in MMTV-erbB2 mice. The results demonstrated that bexarotene significantly prevented the development of premalignant mammary lesions (hyperplasias, DCIS-like lesions, and microscopic invasive breast cancers). Biomarker studies also identified a number of bexarotene-modulated genes both *in vivo* and *in vitro*. Many of these genes play an important role in cell growth regulation. These biomarkers will be further tested in future clinical trials, and will help us to understand the mechanisms by which rexinoids prevent breast cancer.

## MATERIALS AND METHODS

### Transgenic mice, cell lines, and retinoids

All animal studies were performed in an ethical manner and were conducted only after review and approved by the Institutional Animal Care and Use Committee at Baylor College of Medicine. The procedures are in compliance with the United Kingdom Coordinating Committee on Cancer Research guidelines. Female MMTV-erbB2 transgenic mice were obtained from the Jackson Laboratory (Bar Harbor, ME, USA). Mice were housed in the institutional animal facilities and fed a controlled diet of MIN-76A Purified Diet (Harlan Teklad, Madison, WI, USA). Before the treatment, the females received under the right kidney capsule, a pituitary isograft to chronically stimulate the mouse mammary tumour virus (MMTV) promoter (as described by Lydon *et al* (1999)). Virgin animals were used to avoid confounding effects of hormonal surges during pregnancy. Normal human mammary epithelial cells (HMEC) were obtained from Clonetics (San Diego, CA, USA). HMECs were maintained in Mammary Epithelial Basal Medium supplemented with the Mammary Epithelial Growth Media kit (Cambrex Corporation, East Rutherford, NJ, USA). Media were changed every 2 days. Retinoids and rexinoids were obtained from Ligand Pharmaceuticals Inc. (San Diego, CA, USA).

### Treatment and data collection

For short-term treatment, mice were randomised into four experimental groups with 19 mice in each group. The mice were treated with sesame oil or bexarotene (100 mg kg^−1^) for 6 days per week. We previously demonstrated that 100 mg kg^−1^ day^−1^ of bexarotene used in these mice can achieve peak plasma levels that are similar to those from human trials using 200–400 mg m^−2^ day^−1^ (Rizvi *et al*, 1999; Wu *et al*, 2002a). The mice were allowed to age to 3 months before treatment to avoid affecting normal mammary gland development during puberty. Bexarotene was suspended in purified sesame oil (Croda Inc., Mill Hall, PA, USA) and administered by oral gavage using a 20-gauge gavage needle in a volume of 0.1 ml. The mice were killed at the age of 5 months (2 month treatment) or 7 months (4 month treatment). For 2 month treatment, both no. 4 mammary glands were resected, fixed in 4% neutral buffered formalin (4% formaldehyde, phosphate-buffered) overnight and then embedded in paraffin. For the mice treated for 4 months, one no. 4 mammary gland was embedded in paraffin, and the other no. 4 mammary gland was prepared for whole mount analyses. After 4 months of treatment, the no. 4 inguinal mammary glands were harvested, spread onto slides, and fixed with 4% formaldehyde overnight. The glands were placed in 70% ethanol for 1 h and in water for 30 min. The slides were then stained with 0.2% carmine overnight. The mammary glands were dehydrated sequentially in 70, 90, and 100% ethanol for 30 min each, and cleared in toluene to dissolve fat in the gland. The slides were maintained in methyl salicylate for analysis under light microscope.

For biomarker analyses, MMTV-erbB2 mice were randomised into two groups with 20 mice in each group. Mice were treated with vehicle or bexarotene (100 mg kg^−1^) for 6 days per week from 3 months to 5 months of age. At the time of killing, both no. 4 mammary glands were harvested and mammary epithelial pellets were prepared as described (Medina and Kittrell, 2000). Mammary epithelial pellets from four mice were pooled into one sample, and split for RNA and protein isolation.

### Histology and immunohistochemical analyses

Paraffin embedded tissues were sectioned at 4 *μ*m, and processed for hematoxylin and eosin staining for routine histological assessments. Primary antibodies used for immunohistochemical staining include anti-BrdU (clone Bu20a; Dako, Carpinteria, CA, USA), and anti-cleaved caspase-3 (1 : 100, no. 9661, Cell Signaling Technology Inc., Danvers, MA, USA). Briefly, the slides were deparaffinised, and then endogenous peroxidase was blocked in 3% hydrogen peroxide buffer. Samples were incubated with primary antibodies at 4°C overnight followed by incubation with biotinylated rabbit anti-mouse antibody (1 : 100) for 30 min. Peroxidase activity was visualised using Vector NovaRed substrate kit (SK-4800, Vector, Burlingame, CA, USA). The slides were counterstained with hematoxylin for 30 s and then mounted with cover slips.

### Quantitative reverse-transcriptase PCR

Total RNAs were isolated using RNeasy Mini Kit (Qiagen, Valencia, CA, USA). The amount of specific RNA transcripts was assayed by quantitative reverse-transcriptase PCR (QRT–PCR) using gene-specific double fluorescence-labeled probes and an ABI PRISM 7700 Sequence Detector (Applied Biosystems, Foster City, CA, USA). The PCR reaction mixture consisted of 300 nM of each primer, 100 nM of probe, 0.025 U *μ*l^−1^ of Taq Polymerase, 125 *μ*M of each dNTP, 3 mM MgCl2, and 1 × Taq Polymerase buffer. Cycling conditions were 94°C for 1 min, followed by 40 cycles at 94°C for 12 s and 60°C for 30 s. All primers and probes were designed with Primer Express 1.0 software (Applied Biosystems Foster City, CA, USA). 6-Carboxy fluorescein was used as the 5′ fluorescent reporter, whereas blackhole quencher was added to the 3′ end. Standard curves for the quantification of each transcript and cyclophilin were generated using a serially diluted solution of synthetic templates. Genome equivalent copies were calculated from the standard curve and normalised by the amount of cyclophilin. All reactions were performed in triplicate.

### Western blotting analysis

Proteins were isolated by using cell lysis buffer (50 mM Tris-HCL pH 8.0, 2% SDS, and protein kinase inhibitor cocktail). Cell lysates were sheared on ice using a 22G needle and centrifuged at 10 000 × **g** for 30 min. The protein concentration of the supernatant was measured by BCA protein assay (Smith *et al*, 1985). Proteins (30 *μ*g) were resolved on a 10% SDS–polyacrylamide gel. Proteins were then transferred to a nitrocellulose membrane, which was blocked in 5% nonfat dry milk TBST (10 mM Tris pH 8.0, 150 mM NaCl and 0.05% Tween 20) at room temperature for 1 h. Membranes were probed with the primary antibodies in 1% nonfat dry milk/TBST. Membranes were then probed with the corresponding horseradish peroxidase-conjugated secondary antibodies in TBST. The blots were visualised using the ECL Western blot detection system (Amersham Life Sciences, Piscataway, NJ,USA). Involucrin (IVL) and *β*-actin antibodies were obtained from Sigma (St Louis, MO, USA). Differentially Expressed in Chondrocyte 2 (DEC2) antibody was provided by Dr Bingfang Yan.

### Statistical analyses

In the premalignant lesion experiment, numbers of mammary gland showing preinvasive and invasive lesions were counted and analysed by Fisher's exact tests. Immunohistochemical staining of BrdU and cleaved caspase 3 were compared between vehicle and LG100268 groups by Wilcoxon rank sum test. RNA and protein expressions of biomarkers were compared by student's *t*-test (two-tail). *P*<0.05 was considered as statistically significant.

## RESULTS

### Bexarotene prevents the development of premalignant mammary lesions in MMTV-erbB2 transgenic mice

**Figure 1:**
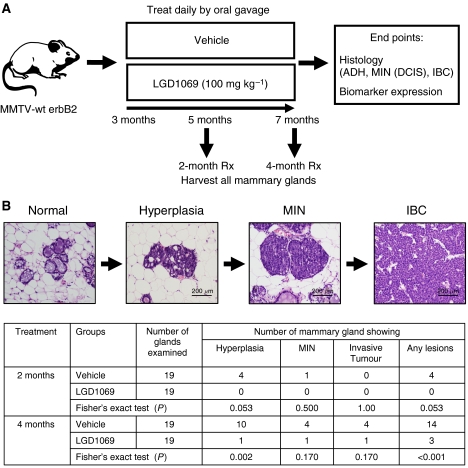
Bexarotene prevents the development of premalignant mammary lesions in MMTV-erbB2 mice. (**A**) Treatment scheme. Beginning at 3 months of age, MMTV-erbB2 mice were treated daily for 6 days per week by oral gavage with either vehicle or bexarotene. The mice were killed after 2 or 4 months of treatment. Mammary tissues were prepared for whole-mount analysis, histology examination, and biomarker measurement. (**B**) Mammary tissue sections were stained with hematoxylin and eosin. For each mouse, we examined one slide from the no. 4 mammary gland for histological analysis. Numbers of mammary gland showing hyperplasias, mammary intraepithelial neoplasias, and invasive mammary tumours were shown. *P*-values were obtained with Fisher's exact tests, generated for each type of lesion individually.

To determine the effect of rexinoids on the development of premalignant mammary lesions, we chose an ER-negative mammary tumourigenesis model that simulates oncogenic events seen in human breast cancer (Hutchinson and Muller, 2000). The tumours arising in these animals show strong similarity to human ER-negative, ErbB2-postitive breast cancer as shown in a recent paper (Herschkowitz *et al*, 2007). MMTV-erbB2 mice carry the unactivated, wide-type erbB2 proto-oncogene whose expression is targeted to breast tissue by the MMTV promoter/enhancer. Similar to carcinogenesis in humans, mammary tumourigenesis in MMTV-erbB2 transgenic mice is a multistage process that proceeds through hyperplasia, mammary intraepithelial neoplasia (MIN, similar to human DCIS), and invasive cancer (Campiglio *et al*, 2004). Pituitary transplants stimulate the transcription of erbB2 gene through activation of the MMTV promoter, and thus promote the development of premalignant and malignant mammary lesions. These mice develop focal tumours beginning at 8 months of age. Therefore, the treatment started at 3 months of age and ended at 5 and 7 months of age (see scheme in Figure 1A), at which time no mice had palpable mammary tumours developed. Normal appearing mammary glands were then removed and processed for histological and biomarker analyses. For these experiments, we examined the frequency of hyperplasias, MIN lesion, and cryptic invasive cancer in these mice. As shown in Figure 1B, at age of 5 months, 21% (4 out of 19) of vehicle-treated mice developed hyperplasias and only one mouse showed a MIN lesion. No invasive breast cancer was detected. In contrast, no hyperplasias or MIN lesions were detected in bexarotene-treated mice. In total there were four animals showing premalignant or cryptic invasive cancers in vehicle-treated mice as compared to none in the bexarotene-treated mice. This difference approached statistical significance (*P*=0.053), but did not reach the cutoff for significance (defined as *P*<0.05). At 7 months of age, we observed an increased numbers of premalignant lesions as well as cryptic microscopic invasive cancers. Compared to vehicle-treated mice, bexarotene-treated mice developed fewer preinvasive and invasive mammary cancers. Specifically, the number of hyperplasias was significantly reduced (*P*<0.01, two-sided Fisher's exact test) by bexarotene treatment. Total number of all lesions was also reduced significantly (*P*<0.01). The number of glands showing MIN lesions or cryptic invasive cancers was also reduced but did not reach statistical significance (*P*=0.17) due to small numbers of lesions. These results suggest that bexarotene prevents mammary tumourigenesis through blocking the development of premalignant lesions.

### Bexarotene inhibits mammary epithelial proliferation

**Figure 2:**
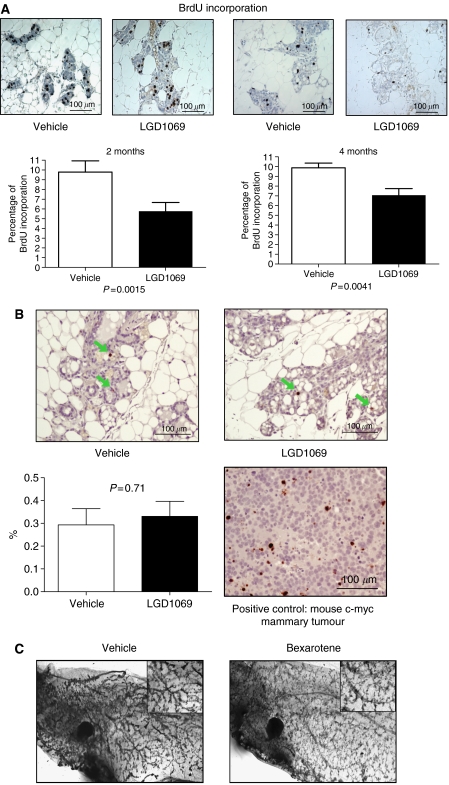
Bexarotene inhibits the proliferation of mammary epithelium. (**A**) Quantitative analysis of BrdU incorporation rate in mammary epithelium from vehicle and bexarotene-treated mice. Mice were treated with vehicle or bexarotene (100 mg kg^−1^) for 2 or 4 months and tissue sections were prepared from paraffin-embedded tissues. Immunohistochemical staining of BrdU was performed as described in ‘Materials and Methods’. Data were generated from 19 animals of each experimental group. Positive stained cells were quantitated from at least 1000 cells. (**B**) Quantitative analyses of cleaved caspase 3 expression in mammary epithelium from mice treated with vehicle or bexarotene for 4 months. Immunohistochemical staining of cleaved caspase 3 was performed as described in ‘Materials and Methods’. Data were generated from 10 animals of each experimental group. Positive stained cells were quantitated from at least 1000 cells. The bars represent s.e. Statistical analyses were assessed with Wilcoxon rank sum test. (**C**) Comparison of mammary gland development in vehicle and bexarotene-treated mice. MMTV-erbB2 mice were treated with vehicle or bexarotene (100 mg kg^−1^) from the age of 3 months to 7 months. Representative whole mount pictures of the no. 4 mammary gland prepared at the end of each treatment are shown.

Previous results have shown that bexarotene significantly inhibits cell growth in normal mammary epithelial cells (Wu *et al*, 2006). Proliferation inhibition and apoptosis promotion are two major causes of growth suppression. To determine which mechanism occurs during bexarotene-induced growth suppression, we examined the effect of bexarotene on proliferation and apoptosis by measuring the levels of BrdU incorporation and cleaved caspase 3. Baseline proliferation of mammary epithelium in MMTV-erbB2 mice was around 10%, higher than the proliferation rate of normal human mammary gland. This is due to the overexpression of erbB2, which stimulates the epithelial proliferation. Bexarotene significantly reduced mammary gland proliferation at both 2 month and 4 month treatments (*P*<0.01, assessed by Wilcoxon rank sum test; Figure 2A). Proliferation was reduced by 40% (10 to 6%) after two months of treatment and by 35% (from 10 to 6.5%) after 4 months of treatment. Next, we determined whether bexarotene induced apoptosis by measuring the proportion of cells staining positive for cleaved caspase 3. As shown in Figure 2B, less than 1% of mammary epithelial cells showed positive caspase 3 staining. There is no statistical difference in the proportion of cells undergoing apoptosis between vehicle and bexarotene-treated mice, suggesting that induction of apoptosis is not responsible for the cancer preventive effect of bexarotene.

### Bexarotene treatment has no effect on mammary gland development

**Figure 3:**
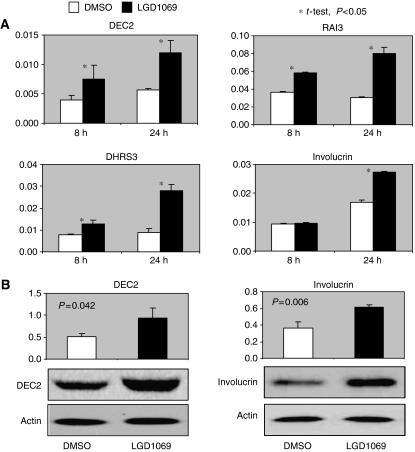
Measurement of rexinoid-modulated genes *in vitro*. (**A**) Measurement of RNA expression. HMECs were treated with DMSO or bexarotene (1 *μ*M) for 8 or 24 h. Total RNAs were isolated. Expression of genes were assessed with quantitative real-time RT–PCR. The expression of each gene was normalised by the expression level of cyclophilin. (**B**) Measurement of protein expression. HMECs were treated with DMSO or bexarotene (1 *μ*M) for 24 h. Total proteins were isolated. Expression of genes was assessed with western blotting. The expression of each gene was normalised by the expression level of *β* actin. The columns represent mean values of three independent experiments. The bars represent s.d. Statistical analyses were assessed with student's *t*-test. DMSO, dimethyl sulphoxide; HMECs, human mammary epithelial cells; RT–PCR, reverse transcriptase-polymerase chain reaction.

To determine whether bexarotene has any effect on mammary gland development, we examined the mammary gland in whole mount after 4 months treatments. As shown in Figure 3C, mammary glands in these mice showed a secretory pattern due to the stimulation from pituitary isograft. When mammary glands from vehicle and bexarotene-treated mice were compared, there was no difference in overall size of the mammary glands or in the number of terminal branches. This result is in contrast to that seen with fenretinide in previous study using a rat model (Radcliffe and Moon, 1983). This may be because we treated mice only after 3 months of age when the mammary glands had fully developed, or it may be due to the specific animal model used in our study.

### Analyses of gene expression in bexarotene-treated breast cells using RT–PCR

As a nuclear receptor ligand, bexarotene exerts its biological activity primarily through modulate the expression of its target genes through RXR. Identification of these genes will help us to understand how rexinoid inhibits cell proliferation and prevents mammary carcinogenesis, and will also identify useful biomarkers for future clinical trials of bexarotene and other rexinoids. We previously have performed Affymetrix Microarray analysis of normal human mammary epithelial cells treated with vehicle or bexarotene (Kim *et al*, 2006). These studies identified many rexinoid regulated genes. Table 1 shows a subset of these rexinoid-regulated genes that were up- or downregulated more than twofold and that were found to show a significant change after a students *t*-test (selected from an initial nonstringent analysis, *P*<0.05). In addition, we also examined DEC genes, which are known retinoid-modulated genes that control cell cycle and proliferation (Boudjelal *et al*, 1997; Azmi *et al*, 2004). We validated the selected gene with reverse transcriptase-polymerase chain reaction (RT–PCR) using independent RNA samples from dimethyl sulphoxide (DMSO) or bexarotene-treated HMEC cells. Among them, 39% (7 out of 18 genes) show significant modulation (*P*<0.05).

### Validation of array data

To further confirm these rexinoid modulated genes, we measured RNA or protein expression levels of selected bexarotene-regulated genes by real-time quantitative RT–PCR and western blotting. Among the 7 genes that had a significant fold change, modulation of ID1, COX-2, and ODC was previously described (Kim *et al*, 2006). We examined another four genes including DEC2, DHRS3, IVL, and RAI3. Three independent sets of total RNA and protein were prepared from DMSO or bexarotene-treated HMEC cells. As shown in Figure 3A, all four genes are modulated by bexarotene significantly after 24 h treatment. We also measured the protein expression of DEC2 and IVL in HMEC cells. As shown in Figure 3B, both genes are induced by bexarotene (*P*<0.05 *t*-test).

### Bexarotene modulates biomarkers in the mammary glands of mice

**Figure 4:**
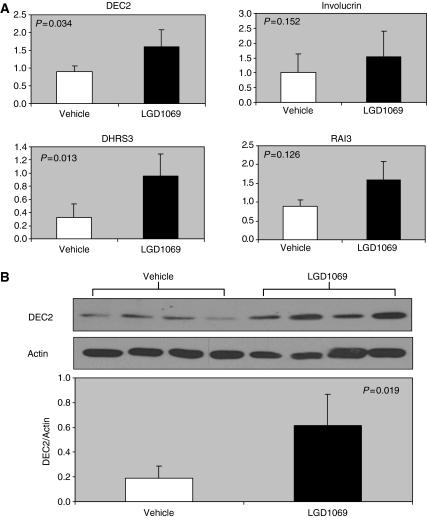
Measurement of rexinoid-modulated genes *in vivo*. MMTV-erbB2 mice were treated with daily oral gavage of vehicle or bexarotene (100 mg kg^−1^) for 2 months starting from 3 months of age. Mammary epithelial pellets were prepared for RNA and protein isolation. Each sample was pooled from four mice. (**A**) Measurement of RNA expression. Expressions of genes were assessed with quantitative real-time RT–PCR. The expression of each gene was normalised by the expression level of cyclophilin. (**B**) Measurement of protein expression. Expressions of genes were assessed with western blotting. The expression of each gene was normalised by the expression level of *β* actin. The columns represent mean values of four pooled samples, as one sample in each group had substantially low yield of RNA/protein and was excluded. The bars represent s.d. Statistical analyses were assessed with student's *t*-test. RT–PCR, reverse transcriptase-polymerase chain reaction.

We have demonstrated that bexarotene regulated specific biomarkers *in vitro* (as shown in Table 1 and Figure 3). Next, we investigated whether bexarotene modulates the expression of these biomarkers *in vivo*. For these experiments, we isolated RNA and protein from mammary epithelial pellets prepared from MMTV-erbB2 mice treated with daily oral gavage of vehicle or bexarotene for 2 months as described in Materials and Methods. At this age, no palpable tumours had developed. The mammary epithelial tissue isolated from the MMTV-erbB2 mice is analogous to breast tissue sample obtained from high-risk women in the clinical chemoprevention studies. As shown in Figure 4A, the RNA expression of four biomarkers (DHRS3, DEC2, IVL, and RAI3) was all modulated by bexarotene in mammary gland cells (although the change in IVL and RAI3 expression did not reach statistical significance). DHRS3 was induced by 300% whereas DEC2 was induced by 79% (*P*<0.05). We also demonstrated that DEC2, a gene that regulates the cell cycle and cellular proliferation, was significantly induced by bexarotene at the protein level in the mammary gland epithelial cells (Figure 4B). The consistency of the *in vivo* and *in vitro* data suggests that these rexinoid-regulated biomarkers will be useful to assess the response to rexinoid treatment in breast cancer prevention clinical trials.

## DISCUSSION

The clinical evidence of fenretinide in preventing secondary breast cancer irrespective of ER status has provided strong rationale to investigate the chemopreventive role of retinoids/rexinoids. We have shown that rexinoid bexarotene is an effective agent to prevent the development of ER-negative breast cancer in MMTV-erbB2 mice. However, the mechanism by with bexarotene prevents tumour development is not fully understood. In this study we investigated the effect of bexarotene on the development of preinvasive mammary lesions. Our results demonstrated that rexinoid bexarotene prevents the development of breast cancer at the early stages during the multistage process of tumourigenesis by suppressing the development of hyperplasias, DCIS-like MIN lesions, and microscopic invasive cancers. Prevention of cancer is associated with reduced proliferation of mammary epithelial cells and with changes in several growth regulating genes. Many of these genes are critical regulators of cell cycle progression and proliferation, and in combination likely mediate rexinoid-induced growth suppression.

Our present results showed that bexarotene suppressed development of hyperplasias, suggesting that this rexinoid prevents the cancer at an early step during tumourigenesis. This is also supported by our published data showing that the proliferation inhibition by bexarotene was more profound in normal mammary epithelial cells than in breast cancer cells (Wu *et al*, 2006). Previous studies showed that bexarotene inhibited the breast cancer cell proliferation *in vitro* (Wu *et al*, 2002a) and carcinogen-inducted mammary tumour growth *in vivo* (Bischoff *et al*, 1999). The antiproliferation effect on both normal and malignant mammary epithelial cells suggests that bexarotene can prevent both the initiation and progression of mammary carcinogenesis. The significant inhibition of mammary epithelial hyperplasias also suggests that the major effect of bexarotene takes places at the early stages during the multistage process of tumourigenesis.

The cancer preventive effects of bexarotene is primarily though proliferation inhibition, as determined by BrdU incorporation rate (Figure 2A). The proportion of cells that undergo apoptosis was not increased after 4 months treatment of bexarotene (Figure 2B). This is consistent with our previous results, in which rexinoid did not promote apoptosis in normal mammary epithelial cells (Wu *et al*, 2006). Christov *et al* (2007) found that short-term treatment with high dose of bexarotene (150 mg kg^−1^) induced apoptosis in methylnitrosourea-induced mammary cancers in rat (Christov *et al*, 2007). However, in MMTV-erbB2 mice, we did not observe apoptosis induction in mammary epithelium at the dose of 100 mg kg^−1^. Sporn's group also found that the rexinoid LG100268 did not induce apoptosis *in vivo* and *in vitro*, although it did synergise with arzoxifene to promote apoptosis (Suh *et al*, 2002; Rendi *et al*, 2004). Recent data showed that both rexinoids (bexarotene and LG100268) significantly downregulate cyclin D1 expression and block cell cycle progression in normal and malignant breast cells (Wu *et al*, 2006). In addition, Yu *et al* (2006) and Landis *et al* (2006) demonstrated that mice lacking cyclin D1 activity were protected from developing erbB2-induced mammary tumours. In this study, we observed a 35–40% of proliferation inhibition by bexarotene. This antiproliferative effect is much more profound than that of gefitinib, which only reduced the mammary epithelial proliferation by 20% (Lu *et al*, 2003). Other investigators have published results showing that bexarotene inhibits proliferation in methylnitrosourea-induced mammary cancers in rats by approximately 75% (Lubet *et al*, 2005; Christov *et al*, 2007). However, the 75% reduction was observed in methylnitrosourea-induced rat tumours, while the 35–40% reduction was observed in normal mammary glands in MMTV-erbB2 mice. We have recently completed a phase II clinical trial using bexarotene to prevent breast cancer in high-risk women at Baylor College of Medicine. The clinical results showed that daily administration of bexarotene (200 mg m^−2^) for 28 days significantly decreased the expression level of cyclin D1 in normal mammary epithelium from post-menopausal women. Bexarotene also decreased the expression of Ki67 by approximately 50% although the result failed to reach statistical significance due to the small sample size (Brown *et al*, 2007). The clinical data support our hypothesis that bexarotene prevents cancer through growth inhibition of mammary gland. These data also suggest that blocking cell cycle progression thorough downregulating cyclin D1 plays critical role in rexinoid-induced proliferation inhibition.

We found that several rexinoid-modulated genes were modulated in the mammary glands from bexarotene-treated mice. These include a metabolism enzyme (DHRS3), differentiation marker (IVL), and genes involved in growth regulation (DEC2, RAI3, ID1, and COX2). Our results show that these rexinoid-modulated genes can be used as biomarkers to demonstrate a biological effect on the target tissue (the mammary gland).

Among the bexarotene modulated genes, DEC2 (Differentially expressed in chondrocytes 1) is particular interesting. Bexarotene induced DEC2 both *in vivo* and *in vitro*. DEC2 is a member of DEC protein subfamily, which consists of DEC1 and DEC2. Both DEC proteins belong to the basic helix-loop-helix transcription factor. DEC1 was originally identified as a retinoid induced gene (Boudjelal *et al*, 1997; Shen *et al*, 1997), whereas DEC2 was first identified through EST analysis (Fujimoto *et al*, 2001). We examined the regulation of DEC proteins and found that DEC2, but not DEC1, was induced by bexarotene. Previous studies showed that DEC proteins are potent transcription repressors by binding to DNA at E-box (St-Pierre *et al*, 2002; Li *et al*, 2003). Azmi *et al* (2003) also found that forced expression of DEC2 inhibited cell proliferation and down-regulated cyclin D1 expression in C2C12 myoblasts. Thus, induction of DEC2 by bexarotene suggests that DEC2 may mediate rexinoid-induced growth suppression.

Our previous studies demonstrated that cyclin D1 was repressed by rexinoids bexarotene and LG100268 (Kim *et al*, 2006; Wu *et al*, 2006; Li *et al*, 2007). Thus, the expression of cyclin D1 is inversely related to DEC2 expression. This inverse correlation raises the possibility that DEC2 might repress cyclin D1. Consistent with this hypothesis, our QPCR data showed that DEC2 was induced by bexarotene at as early as 8 h of treatment. In contrast, downregulation of cyclin D1 by bexarotene was a late effect and was not observed at 8 h of treatment (data not shown), supporting the proposal that DEC2 may play role in suppressing cyclin D1 expression. In addition, another rexinoid downregulated gene, ODC, is known to be regulated by c-myc via an E-box regulatory element. It is possible that DEC2 affects ODC expression in a manner similar to its downregulation of cyclin D1.

These data indicate that rexinoid bexarotene prevents the development of ER-negative mammary tumour at the early stages during the multistage carcinogenesis process in MMTV-erbB2 transgenic mice. The cancer preventive effect is primarily due to inhibition of the proliferation of normal and premalignant breast epithelial cells. We have also shown that rexinoid-regulated genes can be potentially used as biomarkers to show the effects of rexinoids on mammary tissue. These biomarkers will be useful in monitoring the effects of rexinoids in cancer preventive clinical trials. Identification of rexinoid-modulated growth control genes has also helped us in understanding the mechanism by which bexarotene prevents breast cancer.

## Figures and Tables

**Table 1:** Selection of bexarotene-modulated genes by RT–PCR

			**RT–PCR**	
**Gene**	**Full name**	**Array results**	**Fold changes**	***P***
ID1	Inhibitor of DNA binding 1	Up 5.7	↑5.60	0.036
DHRS3	Dehydrogenase/reductase (SDR family) member 3	Up 2.8	↑7.47	0.012
RAI3	Retinoic acid induced 3	Up 3.1	↑3.49	0.042
IVL	Involucrin	Up 2.8	↑2.81	0.049
C-fos	Homologous to the (FBJ) murine osteosarcoma virus oncogene	Up 2.7	↑1.79	0.067
DDIT4	DNA damage inducible transcript 4	Up 4.1	↑1.27	0.285
DSIPI	Delta sleep-inducing peptide, immunoreactor	Up 2.4	↑0.92	0.877
GDI-2	Rho GDP dissociation inhibitor 2	Up 2.0	↑1.20	0.261
DEC1	Differentially expressed in chondrocyte 1		↑1.08	0.795
DEC2	Differentially expressed in chondrocyte 2		↑2.71	0.023
Cox-2	Prostaglandin–endoperoxide synthase 2	Down 4.9	↓3.91	0.045
ASNS	Asparagine synthetase	Down 4.0	↓2.47	0.201
PCK 2	Phosphoenolpyruvate carboxykinase 2	Down 2.0	↓2.42	0.053
TLS/CHOP	TLS–CHOP hybrid gene	Down 4.5	↓1.69	0.091
Wnt7a	Wingless-type MMTV integration site family, member 7A	Down 4.3	↓1.72	0.082
ATF3	Activating transcription factor 3	Down 6.3	↓1.43	0.083
ODC1	Ornithine decarboxylase 1	Down 2.2	↓2.12	0.034
IER2	Immediate early response 2	Down 2.3	↓1.07	0.722

Array results represent the ratio of gene expression in bexarotene *vs* DMSO-treated HMECs determined by an initial nonstringent analysis of Affymetrix microarray (Kim *et al*, 2006). Fold changes represent the ratio of gene expression in bexarotene *vs* DMSO-treated HMECs for 24 h determined by RT–PCR. Whose results were generated from three independent experiments. *P*-values were calculated by using student's *t*-test.
